# Genome-based analysis of the transcriptome from mature chickpea root nodules

**DOI:** 10.3389/fpls.2014.00325

**Published:** 2014-07-11

**Authors:** Fabian Afonso-Grunz, Carlos Molina, Klaus Hoffmeier, Lukas Rycak, Himabindu Kudapa, Rajeev K. Varshney, Jean-Jacques Drevon, Peter Winter, Günter Kahl

**Affiliations:** ^1^Institute for Molecular BioSciences, Goethe University Frankfurt am MainFrankfurt am Main, Germany; ^2^GenXPro GmbH, Frankfurt Biotechnology Innovation Center (FIZ)Frankfurt am Main, Germany; ^3^Plant Breeding Institute, Christian-Albrechts-University KielKiel, Germany; ^4^International Crops Research Institute for the Semi-Arid TropicsHyderabad, India; ^5^French National Institute for Agricultural Research (INRA), Eco&SolsMontpellier-Cedex, France

**Keywords:** *Cicer arietinum*, symbiotic nitrogen fixation, root nodules, chickpea genome sequence, deepSuperSAGE, gene expression profiling

## Abstract

Symbiotic nitrogen fixation (SNF) in root nodules of grain legumes such as chickpea is a highly complex process that drastically affects the gene expression patterns of both the prokaryotic as well as eukaryotic interacting cells. A successfully established symbiotic relationship requires mutual signaling mechanisms and a continuous adaptation of the metabolism of the involved cells to varying environmental conditions. Although some of these processes are well understood today many of the molecular mechanisms underlying SNF, especially in chickpea, remain unclear. Here, we reannotated our previously published transcriptome data generated by deepSuperSAGE (Serial Analysis of Gene Expression) to the recently published draft genome of chickpea to assess the root- and nodule-specific transcriptomes of the eukaryotic host cells. The identified gene expression patterns comprise up to 71 significantly differentially expressed genes and the expression of twenty of these was validated by quantitative real-time PCR with the tissues from five independent biological replicates. Many of the differentially expressed transcripts were found to encode proteins implicated in sugar metabolism, antioxidant defense as well as biotic and abiotic stress responses of the host cells, and some of them were already known to contribute to SNF in other legumes. The differentially expressed genes identified in this study represent candidates that can be used for further characterization of the complex molecular mechanisms underlying SNF in chickpea.

## Introduction

Nitrogen, besides phosphorus and potassium, is one of the primary macronutrients for plants, and consequently a limiting factor for the growth of crops (Crawford, 1995). Although di-nitrogen (N_2_) represents the most abundant gas in the atmosphere, only certain prokaryotes are able to reduce it to an organic form (ammonia), and can thereby make it available for the growth of higher plants. Among these prokaryotes, rhizobia, a class of nitrogen-fixing bacteria, establishes symbiotic partnerships within the roots of some higher plants, which in turn supply them with energy and protect the very sensitive N_2_-fixation machinery from deleterious oxygen. Nutritionally and ecologically seen, symbiotic nitrogen fixation (SNF) identifies chickpea (and other legumes in general) as an important crop, and consequently a promising target for research (Saxena et al., 2012; Varshney et al., 2012). Grain legumes such as chickpea form specialized organs in response to rhizobia invasion, the root nodules. Nodules are highly complex structures that protect the oxygen-sensitive N_2_-fixation machinery from its own byproducts: reactive oxygen and reactive nitrogen species (ROS and RNS, respectively). These are derived from the high rates of respiration, and the leak of electrons from redox proteins to O_2_. As a consequence, nodules are particularly rich not only in quantity, but also diversity of antioxidant defense mechanisms (reviewed in Matamoros et al., 2003), and especially the nodule mitochondria as the principal source of ROS exhibit a comprehensive antioxidant repertoire (Iturbe-Ormaetxe et al., 2001).

Chickpea root nodules are indeterminate in structure, since they maintain a persistent meristematic tissue that produces new cells for growth and renewal of the nodule (Lee and Copeland, 1994). The symbiotic interaction between legumes and rhizobia relies on mutual signal recognition by both partners. Rhizobial signaling molecules called nodulation factors (Nods) are first perceived by cells in the host root epidermis, and induce the expression of early nodulation genes (eNods) in these cells (reviewed in Oldroyd and Downie, 2008). Bacterial invasion of the host cells can occur either through root hair curls or cracks in the epidermis (Gage, 2004). The latter facilitates bacterial invasion of cortical cells, and does not necessarily involve Nod signaling. In general, the formation of indeterminate nodules is accomplished by root hair invasion starting with the adhesion of bacteria to root hairs. Subsequently, the root hairs curl, and the bacteria invade the plant by a newly formed infection thread. At the same time, a nodule primordium is shaped by dividing cortical cells, and once the infection thread reaches the primordium, the bacteria are released into the cytoplasm of the host cells and surrounded by a plant-derived peribacteroid membrane, henceforth termed PBM (Mylona et al., 1995). PBM biogenesis and metabolism is governed by differential gene expression patterns of both the eukaryotic host legume and the prokaryotic rhizobia, which synergistically induce the synthesis of nodulins, bacteroidines, fatty acids, polysaccharides, and other components. The mature PBM provides selectivity for metabolite and ion transport, and facilitates signaling between both the prokaryotic bacteria and eukaryotic plant cells (Krylova et al., 2007). Although these processes are well understood many of the molecular mechanisms underlying SNF in chickpea root nodules remain unclear.

Presently, next-generation sequencing (NGS)-coupled, genome-wide transcriptome profiling techniques represent the principal tools to interrogate the molecular mechanisms of gene expression in organisms across all taxa and in a wide variety of contexts. Especially whole transcriptome shotgun sequencing (RNA-Seq) has recently emerged as a potent technique for transcriptome studies (Libault et al., 2010; Hayashi et al., 2012; Reid et al., 2012; Barros De Carvalho et al., 2013). However, tag-based approaches such as SuperSAGE have several advantages compared to techniques as e.g., RNA-Seq. Transcript abundances are determined more accurately because of the uniform tag length, which impedes an introduction of biases during PCR, and the formation of di-tags, which allows for discrimination of PCR-derived tags. Additionally, the fact that only one tag is generated out of each transcript enables an unequivocal quantification of reads from a given mRNA species, and naturally results in an increased coverage, which facilitates the study of comprehensive transcriptomes as e.g. in plants (Asmann et al., 2009). In line with this, SuperSAGE was applied for the first time to simultaneously assess the differentially expressed genes from the two interacting organisms in *Magnaporthe grisea* (blast)-infected rice leaves (Matsumura et al., 2003). RNA-Seq, on the other hand, provides additional qualitative information, such as isoform expression. The substantially increased sequencing coverage of tag-based approaches must therefore carefully be weighed against the gain of information using whole transcriptome shotgun sequencing.

To date, the adaption of SuperSAGE to NGS, termed deepSuperSAGE, has been used to assess a broad spectrum of transcriptomes in many species (Sharbel et al., 2010; Zawada et al., 2011; Lenz et al., 2013) including our own works on chickpea (Molina et al., 2008, 2011). However, in our previous work on drought- and salinity-stressed chickpea roots, deepSuperSAGE transcription profiling was hampered by the lack of a genomic sequence of chickpea that prevented a faithful functional annotation of many SuperTags. The newly reported drafts of the chickpea genome (Jain et al., 2013; Varshney et al., 2013) finally changed this situation and now enabled us to assign the majority of expressed SuperTags in roots and nodules to a genomic locus and thereby to a potential function in the context of nodulation. The approximately 50,000 duplicate and homopolymer-filtered SuperTags from our previous study in fact represent nearly 1800 genes of which at least 800 are commonly expressed in both tissues. Up to 682 are more abundant in nodule tissue, and 71 genes display a highly significant differential expression (*p*-value < 0.01). The underlying data integrity was previously confirmed via microarray hybridization of approximately 3000 UniTags with diverse regulation levels. Of these, 660 could be reliably measured via hybridization, and the comparison between both platforms resulted in a shared tendency toward up- or downregulation of these transcripts of 79% (Molina et al., 2011). The identified set of differentially expressed genes consequently reflects necessary adaptions of the host cell transcriptome with respect to SNF in chickpea nodules.

In the past, many aspects of nodule development in legumes have been thoroughly characterized (see Ferguson et al., 2010; Desbrosses and Stougaard, 2011; Hayashi et al., 2013). However, less emphasis was paid to the transcriptomes of both nodules and roots, especially in chickpea, since annotation had to be based on the genome sequences of other legumes (e.g., *Medicago truncatula*). Now that the draft genome sequence is available, we reanalyzed the transcriptomes of unstressed chickpea nodule-free roots and mature root nodules from our previous study, and confirmed the newly identified expression patterns via quantitative real-time PCR (qRT-PCR) using five individual biological replicates.

## Materials and methods

### Plant material

Beja 1 (INRAT 93-1) is a salt-tolerant chickpea (*C. arietinum*) variety (L'Taief et al., 2007) that was released in 2003 from the National Institute for Agricultural Research of Tunisia (INRAT) and the International Center for Agricultural Research in the Dry Areas (ICARDA). Rhizobial inoculations and growth conditions for chickpea plants are described in the work of Molina et al. (2011). Briefly, surface-sterilized Beja 1 seeds were germinated on 0.9% agar for five days in a dark chamber at room temperature, and seedlings with a minimum root length of 5 cm were subsequently inoculated with *Mesorhizobium ciceri* strain UPMCa7 (Romdhane et al., 2007). The inoculated seedlings were hydroareoponically grown in a temperature-controlled glasshouse with a day/night temperature regime of 28/20°C, respectively, and a 16 h photoperiod for 40 days. After this period, the six-week-old chickpea plants were harvested from the hydroponic cultures. Nodule tissue was carefully separated from the remaining root system, and subsequently both tissues were immediately stored in liquid nitrogen. Plant breeding was carried out in the greenhouse facilities of the “Soil and Symbiosis Research Unit” of the National Institute for Agricultural Research (INRA) in Montpellier, France.

### Total RNA isolation, library preparation and 454 sequencing

Dissected mature nodules were used in their entirety for characterization of the nodule-specific transcriptome, while transcription profiling of the roots was performed with all the remaining root material available. Total RNA isolation, and subsequent construction and sequencing of SuperSAGE libraries from these tissues were performed as previously described using RNA from a pool of 15 plants (Molina et al., 2011). In brief, total RNA was isolated as described by Pawlowski et al. (1994) with a modified precipitation of the RNA in 3M LiCl at 4°C overnight. Then the polyadenylated fraction of the total RNA was purified using the Oligotex mRNA Mini Kit (QIAGEN, Hilden, Germany), and subsequently used for construction of SuperSAGE libraries as detailed by Matsumura et al. (2003) with a modified sequencing procedure. Instead of di-tag concatenation and subsequent cloning for Sanger sequencing, the PCR amplified di-tags were directly sequenced on the GS20 platform (454 Life Sciences, Branford, USA).

### Bioinformatical processing of deepSuperSAGE sequencing data

Sequencing data was analyzed with the GenXPro SuperSAGE data processing pipeline. First, distinct libraries were sorted out from the bulk of sequences according to their respective barcodes. Then, PCR-derived and all low-complexity reads containing 12 or more consecutive adenine bases were eliminated. These filtered tags were subsequently mapped against the recently published draft of the chickpea genome (Varshney et al., 2013) using the short read mapper Novoalign v2.07.13 (Novocraft Technologies) with default parameter settings. Tags mapping to more than one locus were excluded from further analysis. Finally, feature annotation for the mapped loci was performed with the mRNA sequences from the “Official Gene Set” (OGSv1.0; Varshney et al., 2013), and reads were counted using the Python package HTSeq v0.5.4p2 (EMBL Heidelberg, https://pypi.python.org/pypi/HTSeq). The numbers of unambiguously annotated SuperTags were normalized to 10,000 sequenced tags in total (tags per ten thousand; TPT) for each library to warrant comparability between the libraries. Statistical significance was assessed by χ^2^ tests (Man et al., 2000), and fold changes were determined by pair-wise comparison of the normalized tag numbers. TPT counts of zero were adjusted to 0.05 to allow for calculation of fold changes even if a given tag was only present in one of the libraries (see Table S1).

Functional annotation of the expressed genes was performed with the MapMan software (version 3.5.1) developed by Thimm et al. (2004). First, a reference mapping file comprising a draft metabolic network of chickpea was generated by classification of all protein-coding sequences from the draft genome into MapMan functional categories via the Mercator tool (Lohse et al., 2014). Approximately 60% of these sequences could be assigned to one of the 34 functional classes (Figure S1, Table S2). Subsequently, the generated reference file was used for mapping of the differentially expressed genes onto different metabolic pathways and to assign these genes to several large enzyme families.

### Confirmation of the genome-based reanalysis by quantitative real-time PCR

Quantification of mRNA by real-time PCR was performed on the StepOne Real-Time PCR System (Applied Biosystems) using independent biological replicates that were bred in the same way as described above. Root and nodule tissues from 6 freshly grown plants at the age of six weeks were dissected and used for total RNA isolation with the InviTrap Spin Plant RNA Mini Kit (Stratec Biomedical) following the recommendations of the manufacturer for use of lysis solution DCT. While nodules were simply stripped off the snap-frozen material and afterwards used entirely for total RNA isolation, dissection of root tissue was performed with a sterile razor to obtain absolutely nodule-free root tissue for characterization of the root-specific transcriptomes. Remaining DNA fragments in the isolated total RNA were digested by DNase I (Baseline-ZERO, Epicentre) as recommended by the manufacturer. Subsequent to quantification of the total RNA (Qubit, Life Technologies), all isolates were quality-controlled on the Bioanalyzer 2100 (Agilent Technologies). Isolates with an RNA Integrity Number (RIN) of 7 or higher were reverse-transcribed with SuperScript III Reverse Transcriptase (Invitrogen) following the manufacturer's instructions for first-strand cDNA synthesis. Reverse-transcribed cDNA corresponding to 20 ng total RNA was then added to each amplification reaction on the StepOne Real-Time PCR System. All amplification reactions were carried out in 12 μl volume with the 5x HOT MOLPol EvaGreen qPCR Mix (ROX) from Molegene, complemented by the respective forward and reverse primers (Table 1) in a final concentration of 250 nM each. Initial denaturation was performed at 95°C for 15 min, followed by 40 cycles of 15 s at 95°C, 20 s at 65°C and 30 s at 72°C. A final elongation step at 72°C for 5 min allowed the polymerase to complete all unfinished strands. Subsequently, a melting curve analysis was performed to verify exclusive amplification of the expected products. Additionally, the threshold cycles (*C_t_* values) of the negative (no template) controls from all employed assays were ensured to be higher than 35.

**Table 1:** **List of targeted mRNAs along with the respective primer and probe sequences used for qRT-PCR quantification**.

Gene ID^*^	Alias^*^	**Accession number**^†^	**Primer**	**Sequence**	**Amplicon size**
Ca_04993^+^	Tubulin alpha-7 chain	XM_004501016.1	Forward	GTGGTGATCTTGCCAAGGTTCAG	222
			Reverse	GACTCAGCACCAACCTCTTCATAATC	
Ca_09743^+^	Heat shock protein Hsp90	XM_004491473.1	Forward	TGTTGAAGCTTGGACTGAGCATTG	111
		XM_004491474.1	Reverse	TCGACCTCTTCCATCTTGCTACC	
Ca_03068^+^	S-adenosylmethionine synthase	XM_003609813.1	Forward	CCTCACTATCGTGAAGAACAGCTTTG	166
			Reverse	CCCATTTAAGAGGCTTCACCACTTC	
Ca_06305	Monothiol glutaredoxin-S17	XM_004504992.1	Forward	TGCTCCAAGATGCGGCTTTAG	187
			Reverse	CCATAACAATATCGCAACCGCCTATC	
Ca_13049	Integrator complex subunit	XM_004512818.1	Forward	AAATTGCAGCTGATTTGGCTTCC	302
			Reverse	AAGCTTTGTAAGGGTCCTCTGTATG	
Ca_22734	Putative uncharacterized protein	XM_004512804.1	Forward	CAATGAAGCGTTCGGGTTTGTG	114
			Reverse	CTCCGACCGCCACAACATATC	
Ca_10340	Multidrug resistance protein	XM_004503208.1	Forward	AGAGTCAGGGCATGACACTCATC	148
		AB024992.1	Reverse	CCGTGCCATGGGATGCTTAG	
Ca_04229	Neutral alpha-glucosidase AB	XM_004502926.1	Forward	GGCACCTACTTCTGGTGGAAATG	169
			Reverse	AAGCTGGTGAATGTGCCCTTTG	
Ca_07680	Putative uncharacterized protein	XM_004494957.1	Forward	CAGGAAACAGCCGAAATCTAGGATG	132
			Reverse	ACAAGCTTCTGGCCAACTATTGC	
Ca_06862	Putative uncharacterized protein	XM_004508211.1	Forward	GCTGTCCATGAGAAAGGAGATGTG	142
			Reverse	AGCTGCTCTTGGAAACTGCTTTG	
Ca_11013	Putative uncharacterized protein	XM_004498494.1	Forward	ATGGTGCACATGGAGAATTCATGG	242
			Reverse	GCAACATAGGAAGCCCTGCATAG	
Ca_10312	Coronatine-insensitive 1	XM_004503173.1	Forward	AGGGTATGGTGCATCTCCATCTG	181
			Reverse	AATCTGATCTTTGGCCAGCAAGAG	
Ca_16834	HMG I/Y like protein	XM_004512669.1	Forward	AACAACACCTGCTAGTGCTCAAC	110
			Reverse	AAATGAGGCCTAAGCACTGCAAG	
Ca_05800	6-phosphogluconate dehydrogenase	XM_004503591.1	Forward	CTTGTTCAGGCTCAGAGGGATTTG	126
			Reverse	AATTAAGAGCAGCAACACCAGTACC	
Ca_22023	Monosaccharid transport protein	XM_004504903.1	Forward	CATGTTGCCTGAGACTAAGGGAATAC	133
			Reverse	TAACAGCTCCCTTGCCCATCTC	
Ca_00007	Squamous cell carcinoma antigen	XM_004485354.1	Forward	TTTGGACGATGAGCACCTTGTTG	225
		XM_004485355.1	Reverse	AATGCTCTCACTTCGTGGCTTTC	
		XM_004485356.1			
		XM_004485357.1			
Ca_05370	Prefoldin subunit	XM_004497178.1	Forward	CTCAACACGTTCTCGTCGATGTC	134
		XM_004497179.1	Reverse	TTTGGGATGCCACCTCAACAAG	
		XM_004497180.1			
Ca_15777	Serine/threonine protein kinase-like protein CCR4	XM_004506314.1	Forward	TGGACCCTGAATACTATAGGCTACAAC	332
			Reverse	ACGCCGCAAGAGCTGTTTC	
Ca_12354	ADP-ribosylation factor GTPase-activating protein	XM_004509671.1	Forward	TTCCATCTCCAGTGCCGATCTC	200
			Reverse	CAGAGAATTCGGTCTTGAAGATCTGTC	
Ca_15466	Nodulin 6	XM_004497937.1	Forward	ACTGATGCCTATGCATTTCCTGAAC	124
			Reverse	ACTTCCACAGCCTCCGGAAC	
Ca_12714	Putative uncharacterized protein	XM_004502350.1	Forward	GGCCAATCCTGAGAAGAGAATCAC	229
		XM_004502351.1	Reverse	CCATGCCTCCTCCAACAAATTGTC	
Ca_13139	Putative uncharacterized protein	XM_004498271.1	Forward	TGGCTGAACAAACTCATTTGGGAAG	219
		XM_004498272.1	Reverse	CCTGCAACCTTGATATCTCCAGGAAC	
Ca_03442	Glutathione S-transferase	XM_004495920.1	Forward	GGAAGAGAATGAAGCCAAGTTGAACAC	217
			Reverse	TAGACCAAGCTGGTCTTGCAGTG	
Ca_16084	Leghemoglobin	XM_004490852.1	Forward	GAGATGCTACATTGGGTGCTGTTC	159
			Reverse	GCCAATCCATCATAGGCGAGTTC	

* Gene ID and alias according to OGSv1.0 (Varshney et al., 2013);

† NCBI reference sequences for all transcript variants targeted by the respective primer pair;

+ reference gene used for normalization.

The relative transcript abundances between root and nodule tissue from the different biological replicates were calculated according to the ΔΔ*C_t_* method using the geometric mean of three previously determined reference genes. All target and reference mRNAs were quantified in duplicates for all biological replicates, respectively. The arithmetic mean of each duplicate was then used to calculate the ΔΔ*C_t_* values between the tissues. Thirteen candidate reference genes were screened for their target stability using a pool of reverse-transcribed cDNAs from the five biological replicates that passed quality control (Figure S2A). The determined expression ratios were analyzed with geNorm (Vandesompele et al., 2002), and six of the best performing candidates (geNorm M < 0.5) were analyzed in more detail using three individual biological replicates (Figure S2B). The optimal number of target reference genes was determined to vary around two to three. To ensure optimal comparability, the 3 best-performing candidates from the individual test run (Ca_04993, Ca_09743, and Ca_03068) were subsequently used for normalization of the gene expression ratios from root and nodule tissue.

## Results and discussion

### Genome based reanalysis

**Figure 1:**
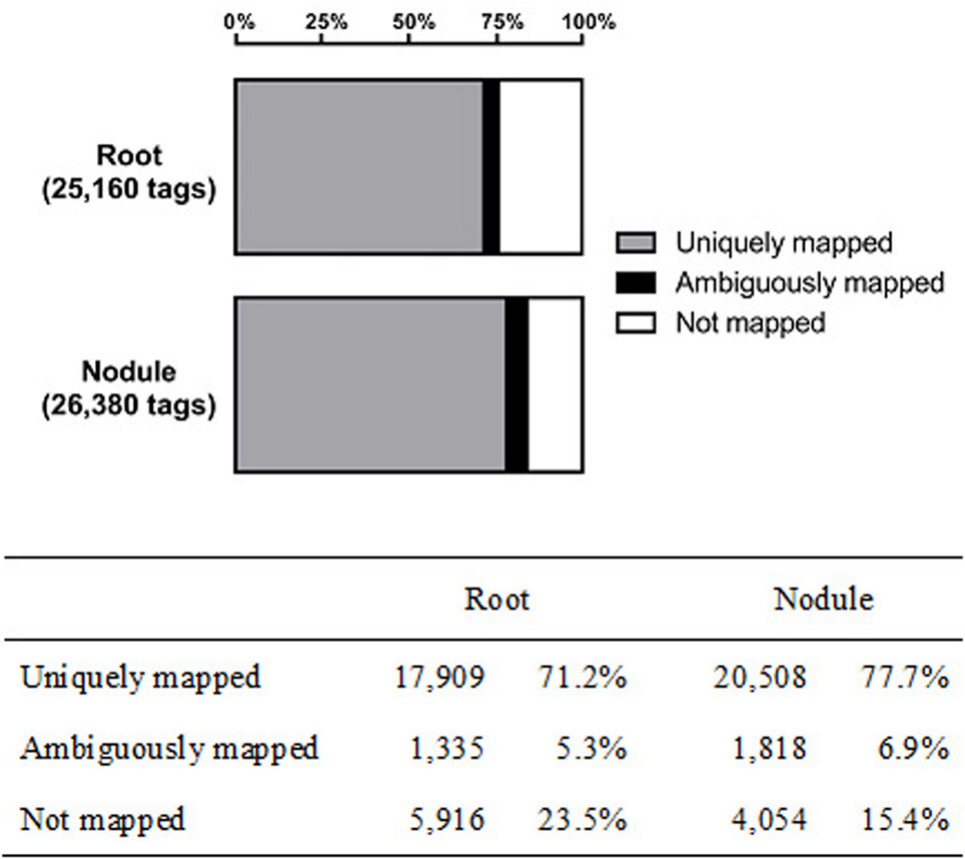
**Genomic mapping results of the captured transcriptomes from chickpea root and nodule tissue**.

With a duplicate- and homopolymer-filtered read number of 25,160 (roots) and 26,380 (nodules), both libraries contain a similar number of reads (±5%). Genomic mapping resulted in 17,909 (71.1%) and 20,508 (77.7%) unambiguously assigned tags (root and nodule, respectively). The remaining reads were either mapped to more than one locus or could not be mapped to the mRNA-encoding sequences of the chickpea genome at all (Figure 1). Only uniquely assigned reads were taken into consideration for further analysis.

In our study in 2011, the 51,545 sequenced Beja 1 SuperTags from root and nodule tissue of unstressed chickpea plants were grouped into 11,525 UniTags (SuperTags of common origin), while the genome-based reanalysis of these SuperTags resulted in the identification of approximately 1780 expressed gene loci. These represent the transcriptionally most active genes in chickpea roots and nodules, and consequently the corresponding mRNAs are the most abundant transcripts. The 140 UniTags previously found to be more than 8-fold prevalent in nodules were reduced to 61 significantly differentially expressed loci in the genome of chickpea (Table 2). On the other hand, the number of more than 20-fold differentially expressed UniTags between both tissues increased from four transcripts to 64 that actually exhibit more than a 128-fold differential expression. Consequently, many of the slightly upregulated UniTags in nodules are expressed from the same genomic locus, and based on the genome sequence these can be combined to provide more meaningful data.

**Table 2:** **Number of differentially expressed genes in root and nodule tissue from chickpea cultivar Beja 1**.

	**Differentially expressed**					
	**>2-fold**	**>4-fold**	**>8-fold**	**>64-fold**	**>128-fold**	**>256-fold**
Nodule upregulated	108 (953)	92 (755)	61 (692)	51 (296)	51 (51)	5 (5)
Nodule downregulated	56 (228)	42 (159)	26 (135)	13 (50)	13 (13)	3 (3)
Sum of differentially expressed genes	164 (1181)	134 (914)	87 (827)	64 (346)	64 (64)	8 (8)

Numbers on the left represent significantly differentially expressed genes (α = *0.05*), while the numbers in brackets include all expressed genes regardless of any significance threshold.

### Nodule-specific gene expression of chickpea cultivar Beja 1

**Figure 2:**
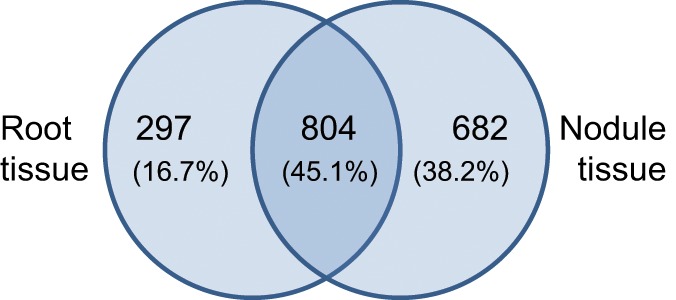
**Tissue-specific gene expression in chickpea cultivar Beja 1.** Outer numbers represent genes that are expressed in root (left) or nodule (right) tissue, while the number within the overlap represents commonly expressed genes.

The identified expression profiles of Beja 1 root and nodule tissues share around 800 (~45%) expressed genes (Figure 2), while 682 (almost 40%) of the genes are heavily upregulated in nodule tissue (vs. 297 distinctly expressed genes in roots). Although the captured number of expressed genes (~1780) is relatively low compared to more recent profiling studies, the large difference in distinctly expressed genes indicates important variations in expression of the most abundant transcripts in the context of nodulation. The relatively high number of expressed genes in nodule tissue reflects an induced expression of a plethora of genes that are putatively involved in establishment and maintenance of the symbiotic relationship. A total of 71 genes were identified as highly significant (α = 0.01) differentially expressed between both tissues (Table S1). The distribution of these genes across the 8 chickpea chromosomes is relatively uniform and varies around nine (±50%) differentially expressed genes per chromosome. With respect to the varying chromosome sizes, most of the differentially expressed genes are located on chromosome 8, but no particular chromosomal region was found to be significantly enriched with differentially expressed loci. Genes showing more than a 2-fold differential expression between roots and nodules are listed in Table 2. Consistent with the high number of expressed genes in nodule tissue, the number of upregulated genes in nodules is about twice as high as in roots. As expected, most genes display a relatively low differential expression, and therefore the respective significance levels vary accordingly. Sixty four significantly differentially expressed transcripts (enrichment of 128-fold or more) could be identified as highly enriched in one of the tissues. Among others, these genes include BZIP transcription factor 2 (inferred from *Phaseolus vulgaris*), nodulin 6, abscisic acid receptor PYL4, and glutathione S-transferase (inferred from *M. truncatula*) all of which are upregulated in nodules (Table S1). The complete set of expressed genes is depicted in a heat map (Figure S3) that illustrates the relatively high expression ratio in nodule compared to root tissue, since numerous transcripts are found to be more abundant in nodules.

### Validation of the identified gene expression patterns by quantitative real-time PCR

**Figure 3:**
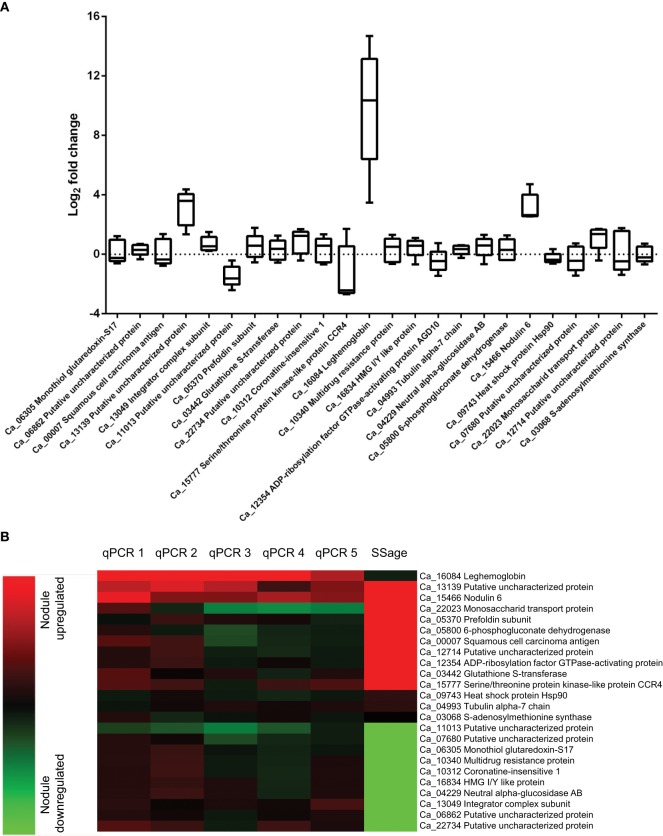
**Biological variance in gene expression of 21 candidate and three reference genes across five biological replicates (A) and comparison of deepSuperSAGE expression patterns from ten pooled plants with the individual expression ratios determined by qRT-PCR (B).** The logarithmized (base 2) expression ratios between root and nodule tissue from five biological replicates are depicted in box plots for the indicated genes. Positive values represent nodule upregulated mRNAs and negative values mRNAs that are more abundant in root tissue. The gene expression ratios of the individual biological replicates (qPCR1-5) in comparison to the pooled (SSage) tissues are additionally shown in the heat map. Nodule upregulated transcripts are represented in red and downregulated transcripts in green. Clustering was performed with the MultiExperiment Viewer version 4.9 by hierarchical clustering of all genes and samples using Euclidean distance calculations.

Differential expression of the ten most up and downregulated genes in chickpea nodule tissue from five biological replicates was validated via qRT-PCR and revealed a comprehensive biological variance between the different isolates (Figure 3A, Table S1). While all of the upregulated and seven out of the ten most downregulated genes in nodule tissue are found to be accordingly expressed in at least one of the replicates, some of these genes exhibit converse expression ratios in the other replicates. The expression of leghemoglobin, which is known to be expressed by legumes in response to colonization of the roots by rhizobia (Benedito et al., 2008; Libault et al., 2010), was assessed additionally to the twenty most differentially expressed genes. As expected, the mRNA encoding leghemoglobin is significantly more abundant in nodule tissue regardless of the biological replicate. The individual expression ratios, however, range from almost 25,000-fold down to 10-fold upregulated in the respective tissues. The extensive biological variance in expression of some of the twenty candidate genes becomes even more apparent after hierarchical clustering (Figure 3B). The individual replicates can be broadly classified into two groups that show similar expression ratios either for the upregulated (qPCR1 and qPCR2) or alternatively for the downregulated transcripts (qPCR3-5) compared to the pool of ten plants used for deepSuperSAGE.

### Upregulated genes in chickpea nodules

The 10 most upregulated genes in chickpea nodule tissue are listed in Table 3. The proteins encoded by two of the listed genes have a putative function, but the respective protein sequences could be matched with the InterPro database, which predicts a serine/threonine-protein kinase and a drug/metabolite transporter. Especially the transcript encoding the putative drug/metabolite transporter is highly upregulated in nodules (>300-fold enrichment) not only in the pooled plant tissue but also in the individual biological replicates, which suggests that the corresponding gene product is functionally important in the rhizobia-adapted metabolism of nodules. The putative drug/metabolite transporter (IPR000620) is predicted to be an integral membrane protein and may contribute to the selectivity of metabolite transport through the mature PBM.

**Table 3:** **Proteins encoded by the 10 most upregulated genes from nodule compared to root tissue of chickpea cultivar Beja 1**.

**ID**	**TrEMBL database**	**InterProScan**	**Fold change**	***P*-value**	**Chr.**
Ca_13139	Putative uncharacterized protein	Drug/metabolite transporter	346	0.0008	4
Ca_03442	Glutathione S-transferase	Glutathione S-transferase	346	0.0008	4
Ca_12714	Putative uncharacterized protein	Protein kinase, catalytic domain; Serine/threonine-protein kinase domain	311	0.0015	5
Ca_12354	ADP-ribosylation factor GTPase-activating protein AGD10	Arf GTPase activating protein	276	0.0028	7
Ca_15466	Nodulin 6	Amidohydrolase 2	276	0.0028	4
Ca_15777	Serine/threonine protein kinase-like protein CCR4	Protein kinase, catalytic domain; Serine/threonine-protein kinase domain	242	0.0051	6
Ca_05370	Prefoldin subunit	Prefoldin subunit	242	0.0051	4
Ca_05800	6-phosphogluconate dehydrogenase, decarboxylating	6-phosphogluconate dehydrogenase, C-terminal; NAD-binding	207	0.0095	6
Ca_22023	Monosaccharide transport protein	Sugar/inositol transporter	207	0.0095	6
Ca_00007	Squamous cell carcinoma antigen recognized by T-cells, putative	RNA recognition motif domain; RNA-processing protein, HAT helix	207	0.0095	1

Protein functions inferred from the TrEMBL and InterPro database are listed along with the respective fold changes, p-values (χ^2^) and chromosomal positions. This list represents an excerpt from Table S1.

Another strongly upregulated transcript in nodule tissue encodes a glutathione S-transferase (GST) that is implicated in the antioxidant defense of legume root nodules. GSTs can directly scavenge peroxides with the help of glutathione as electron acceptor, and furthermore detoxify endogenous compounds such as peroxidized lipids via conjugation of glutathione to target substrates, which facilitates their sequestration and removal (reviewed in Becana et al., 2010). Not surprisingly, GSTs constitute a large gene family in legumes, whose N_2_-fixation machinery needs protection from ROS and RNS. Nodulin 6, encoded by early nodulin gene *MtN6* in *M. truncatula*, is one of the early response genes upon rhizobia infection in legumes. The mRNAs coding for Nodulin 6 accumulate in outer cortical cells that contain pre-infection threads, and in front of growing infection threads in mature root nodules (Mathis et al., 1999). Upregulation of Nodulin 6 consequently reflects the ongoing root hair invasion in mature nodule tissue.

The transcript encoding 6-phosphogluconate dehydrogenase (6PGD) is more than 200-fold upregulated in the pooled nodule compared to root tissue. The encoded enzyme belongs to the family of oxidoreductases and is involved in the non-oxidative phase of the pentose phosphate pathway, where it catalyzes the conversion of 6-phosphogluconate to ribulose 5-phosphate. The upregulation of *6PGD* resonates with the induced expression of ribulose-phosphate 3-epimerase (~170-fold upregulated in nodules, Table S1), which in turn uses ribulose 5-phosphate as a substrate to generate the ketose sugar xylulose 5-phosphate. A primary end product of the pentose phosphate pathway is NADPH, which is needed in response to oxidative stress, besides being necessary for fatty acid synthesis. NADPH can serve as co-substrate for glutathione reductases that reduce oxidized glutathione e.g. subsequent to its oxidation via GSTs (Table 3) or other glutathione peroxidases (up to 3-fold upregulated in nodules, Table S1). Further end products of the pathway include ribose-5-phosphate, used in the synthesis of nucleic acids, and erythrose-4-phosphate, implicated in the synthesis of aromatic amino acids. Against this backdrop, the induced expression of several members of the pentose phosphate pathway is not exclusively linked to an increased anabolism in nodule tissue, but rather ensures a steady supply with reducing equivalents (in form of NADPH).

The heavily upregulated monosaccharide transport protein (MTP) represents an ortholog of the hexose transporter Mtst1 in *M. truncatula*. An alignment of the mRNA sequence of Mtst1 from *M. truncatula* with the genomic sequence of *MTP* resulted in 44% matching bases, although the *MTP* sequence still retains introns (Figure S5). Interestingly, expression of Mtst1 was associated with a successfully established symbiosis of *M. truncatula* and the vesicular-arbuscular mycorrhizal fungus *Glomus versiforme* (Harrison, 1996). *In situ* hybridization revealed high Mtst1 mRNA levels in phloem fiber cells of the vascular tissue, cells of the root tip, and in cortical cells of the mycorrhizal root, especially in highly invaded areas. The author therefore suggests that increased expression levels of Mtst1 are correlated with internal growth of the fungus and with a functioning symbiosis, because the affected cells exhibit an increased metabolism, which in turn requires an intensified energy supply. To our knowledge an ortholog of Mtst1 in chickpea has not yet been described. Analogous to the function of Mtst1 in the context of vesicular-arbuscular mycorrhizal associations, MTP potentially ensures the sugar supply for root cells directly involved in the symbiotic association of rhizobia and legumes. The strong upregulation of *MTP* in nodules might furthermore contribute to the salt-tolerant trait of chickpea cultivar Beja 1, since hexose sugars also increase the cytosolic solute concentration, and thus can contribute to maintain the osmotic pressure (Hasegawa et al., 2000). One of the major bottlenecks of SNF in plants is the sensitivity of the symbiotic interaction, which renders the nodules very susceptible to abiotic stresses. The activity of enzymes directly involved in SNF, for example, was drastically decreased in salt-stressed nodules (Cordovilla et al., 1994). The nodule-specific induction of *MTP* along with *6PGD* thus indicates that several of the upregulated genes in nodules are not only linked to an increased metabolism, but also involved in the salt-tolerant trait of Beja 1. This is in line with our previous finding that several relatively low expressed UniTags in unstressed nodules become highly abundant in salt-stressed nodule tissue (Molina et al., 2011). 6PGD was additionally identified as a stress-responsive protein in a comparative proteomic analysis of *A. thaliana* roots that had been exposed to 150 mM NaCl (Jiang et al., 2007). The differential expression of this gene with the onset of salt stress in *A. thaliana* seems to be tightly linked to the accumulation of compatible osmolytes, and the present findings emphasize the importance of this mechanism in the context of SNF in legumes.

### Functional classification of differentially expressed genes in chickpea nodule tissue

The identified expression patterns of chickpea root and nodule tissues display comprehensive differences. Many gene products involved in sugar metabolism, antioxidant defense as well as biotic and abiotic stress responses of the host cells show drastically altered levels after adaption to the symbiotic relationship (Figure S4). This is in line with a recent report of nodulation-relevant genes identified in soybean roots 10 days after inoculation with *Bradyrhizobium japonicum* (Barros De Carvalho et al., 2013). The upregulated genes in nodules were found to be primarily involved in host cell metabolism, cell wall modifications and the antioxidant defense system. Interestingly, glycolysis and the citric acid cycle were the most active metabolic pathways in the context of SNF. The strong induction of several members of the pentose phosphate shunt in Beja 1 root nodules, however, reflects a shifted nitrogen balance in nodule tissue that is linked to an increased anabolism. Naturally, the interacting host cells have to ensure a steady supply of metabolites for the nitrogen-fixing rhizobia on the one hand, but also provide nitrogenous compounds for the rest of the plant on the other. Accordingly, the gene encoding asparagine synthetase is heavily upregulated in nodule tissue (~70-fold, see Table S1, Figure S4B), since asparagine represents one of the primary nitrogen transport products in plants.

**Figure 4:**
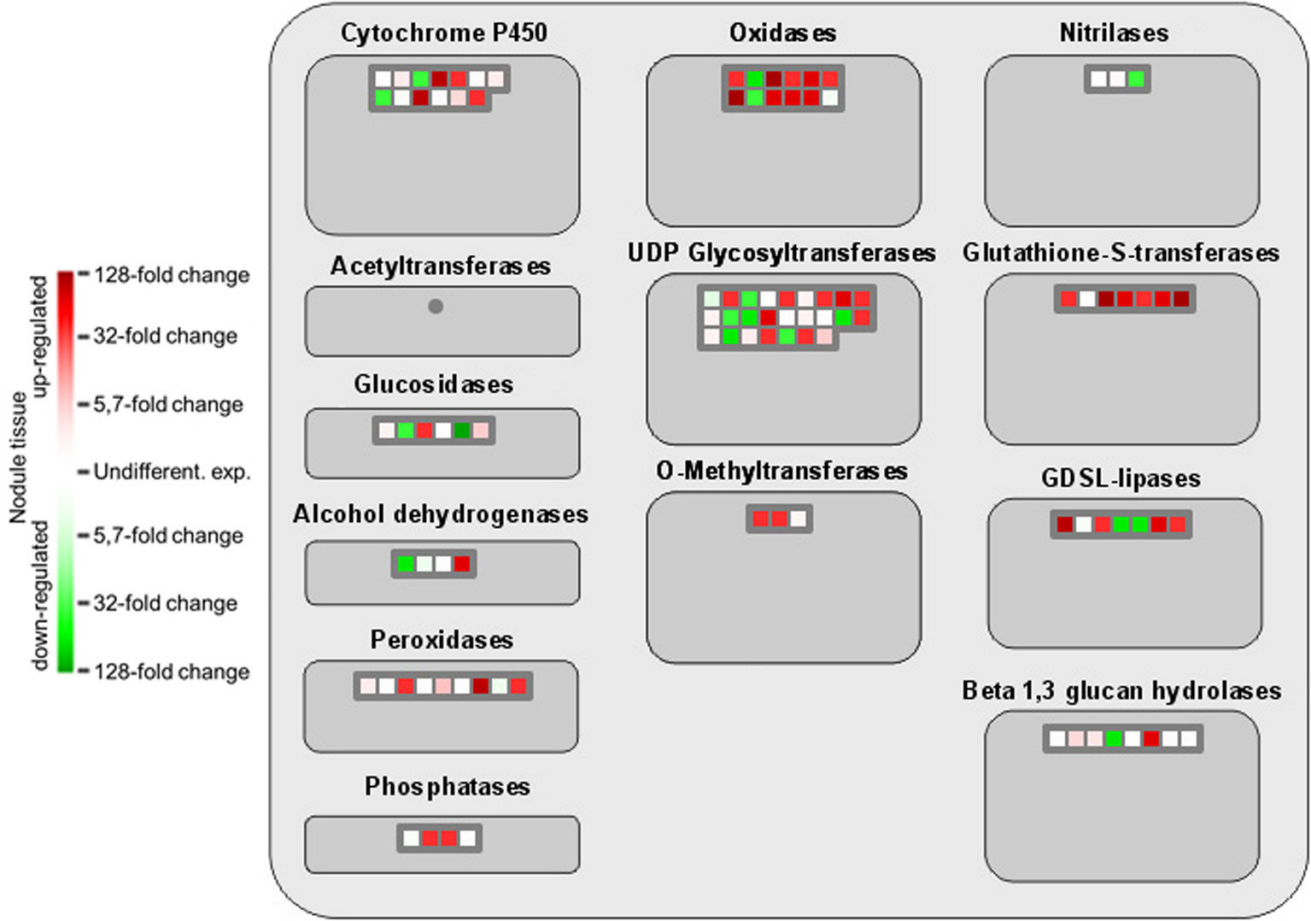
**MapMan-based classification of the expressed genes into large enzyme families.** Each field represents the expression of a particular gene. Upregulated genes in nodule tissue are shown in red, genes with reduced expression in green, and undifferentially expressed ones in white. Dark gray fields indicate that none of the expressed genes could be assigned to the respective class.

The comprehensive expression of genes encoding GSTs in nodule tissue is accompanied by an upregulation of several oxidoreductases involved in different metabolic pathways as well as the two isoflavone 7-O-methyltransferase isoforms 8 and 9 (Figure 4). A globally induced expression of genes encoding GSTs was previously identified in nodule tissue from *M. truncatula* by Benedito et al. (2008), and the present findings confirm the importance of ROS scavenging for SNF in chickpea. Interestingly, overexpression of isoflavone 7-O-methyltransferase 8 was shown to enhance disease resistance in *M. sativa* (He and Dixon, 2000), while the other isoform was identified as drought-responsive transcript in chickpea (Varshney et al., 2009). Against this backdrop, upregulation of isoform 9 could well contribute to the salt-tolerant trait of Beja 1, since an *a priori* increased expression of stress-responsive genes already prepares the very sensitive interaction of eukaryotic and prokaryotic cells in root nodules for a possible onset of stress. Expression profiles of other large enzyme families such as nitrilases, glucosidases or the cytochrome P450 superfamily of monooxygenases are less consistent, but several peroxidases implicated in the response to oxidative stress as well as defense responses to fungi are heavily upregulated in nodules. These peroxidases apparently add to the comprehensive antioxidant defense repertoire that protects the oxygen-sensitive N_2_-fixation machinery in chickpea nodules.

**Figure 5:**
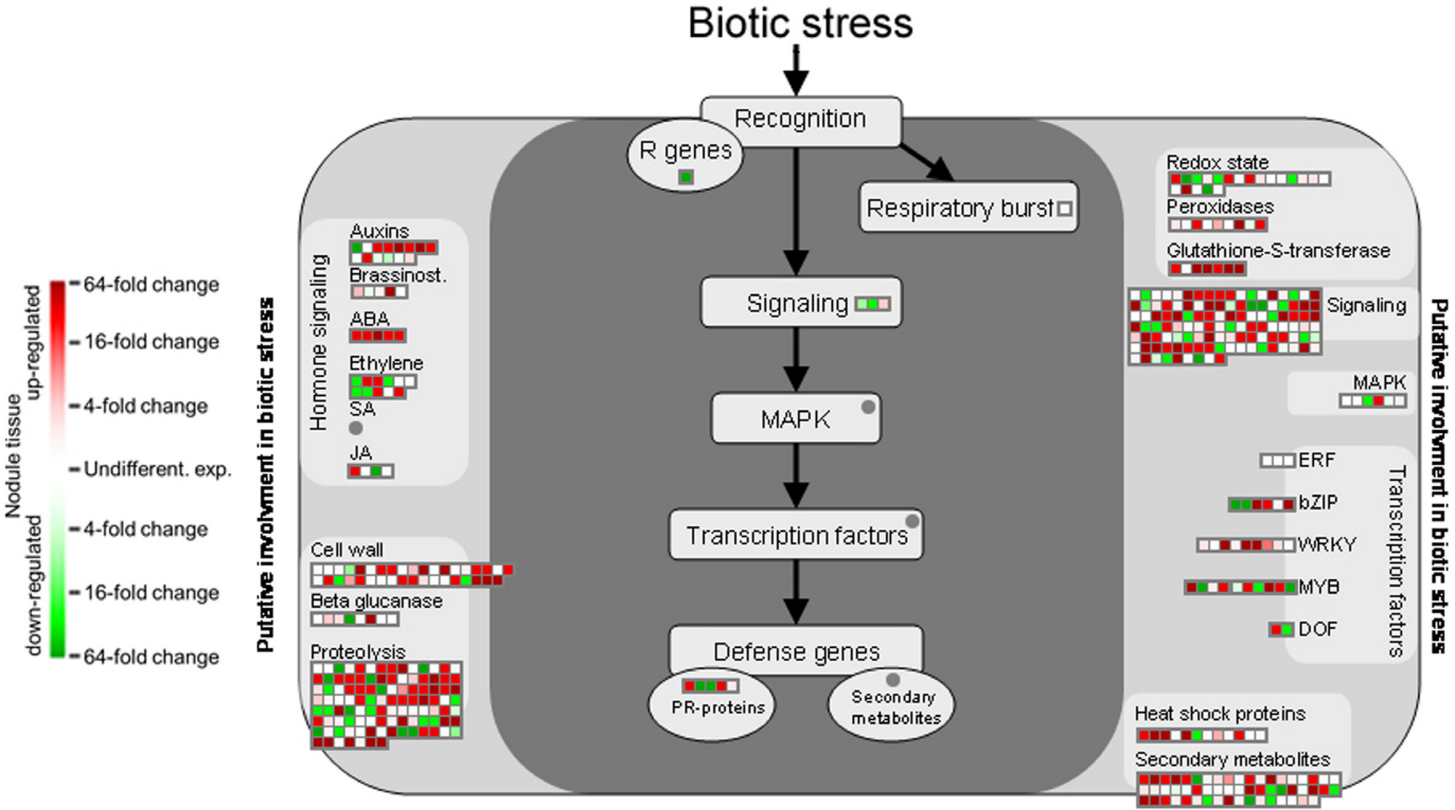
**Gene expression relating to biotic stress pathways based on the functional annotation with MapMan.** Please consult Figure 4 for further details.

Functional annotation onto known biotic stress pathways reveals a reduced expression of disease resistance proteins, defense genes and of genes involved in mediating host cell responses to pathogens (Figure 5). This is consistent with the massive downregulation of the multidrug resistance and disease resistance response proteins in mature Beja 1 nodules, and confirms our previous observation that a sustained inhibition of at least some of the defense reactions in root nodules seems to be a prerequisite for maintenance of the symbiosis.

**Figure 6:**
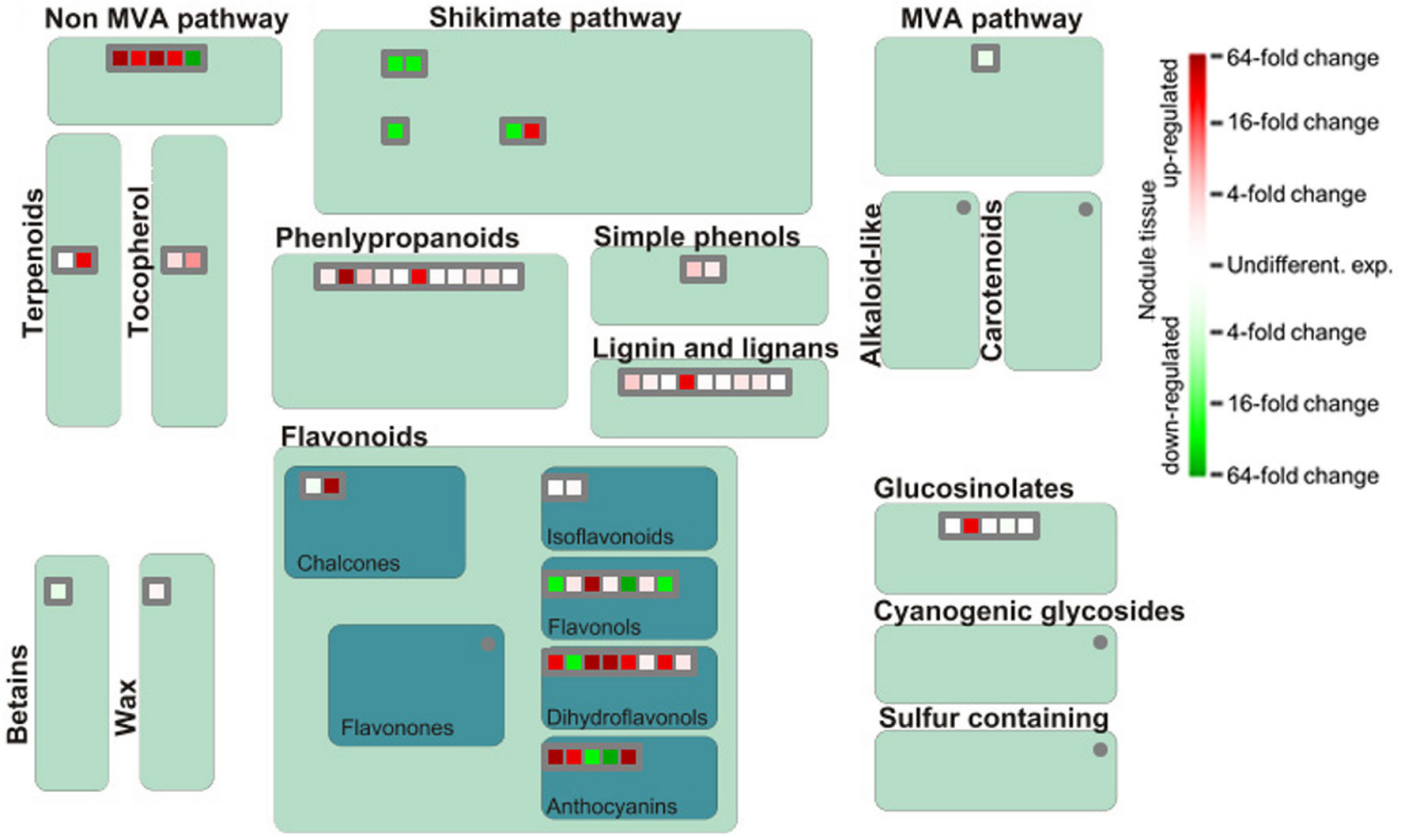
**Differential expression of genes involved in biosynthesis of secondary metabolites.** Please consult Figure 4 for further details.

The functional analysis of genes involved in generation of secondary metabolites in nodules illustrates a strong upregulation of genes implicated in phenylpropanoid and flavonoid biosynthesis although several genes encoding proteins of the shikimic acid pathway are downregulated (Figure 6). Especially dihydroflavonols appear to be of special importance for SNF, which is likely linked to their function as antioxidants and radical scavengers (Haraguchi et al., 1996). The most upregulated gene involved in the synthesis of phenylpropanoids actually encodes a nicotinamidase that operates in the salvage pathway of NAD biosynthesis (Wang and Pichersky, 2007). The induced expression of this gene in nodule tissue underlines the importance of NAD as redox carrier in the rhizobia-adapted metabolism.

## Conclusions

The increasing throughput of next-generation sequencing technologies and the application of 3rd generation sequencing (such as the SMRT sequencing system from Pacific Biosciences) are currently intensifying the efforts to decipher the genome of a steadily increasing number of legumes. As in the case of chickpea, these genome sequences will allow for more detailed analyses of the differential gene expression in relation to SNF in legumes. Using the recently published chickpea genome sequence, we reanalyzed the transcriptomes of unstressed root and nodule tissue and identified a strong upregulation of genes encoding glutathione S-transferases or with implications in phenylpropanoid and flavonoid biosynthesis. The expression of twenty candidate genes from the reanalyzed dataset was validated using five individual biological replicates, revealing a comprehensive biological variance in the corresponding expression. Additionally to the characterization of the most differentially expressed mRNAs and their potential functions in relation to SNF, we implemented a functional analysis via MapMan for the transcriptomes of both tissues. The functional classification of all protein-coding chickpea genome sequences will pave the way for future functional analyses of chickpea mRNAs, also retrospectively (as in the present study) and not only from root or nodule, but from any tissue of interest.

### Conflict of interest statement

The authors declare that the research was conducted in the absence of any commercial or financial relationships that could be construed as a potential conflict of interest.
