# Supplementary material for: Genome-based analysis of the transcriptome from mature chickpea root nodules
Source: Front Plant Sci. 2014 Jul 11;5:325. doi: 10.3389/fpls.2014.00325 (PMC4093793; doi:10.3389/fpls.2014.00325)
Supplement: Supplementary file 1 [file Presentation1.ZIP › Supplementary Material legends.docx]

# Supplementary material

Table S1 | Complete data matrix

Table S2 | Reference mapping file for functional annotation via MapMan

Figure S1 | Results of the functional classification for the reference mapping file using Mercator. The percentage of assigned reads for each of the 34 functional classes is listed and depicted in a circle graph along with the numbers of unassigned reads.

Figure S2 | Reference target stability of selected candidate genes according to geNorm. The average expression stability (geNorm M) of 13 potential reference genes in a pool of reverse-transcribed cDNAs from root and nodule tissue of five biological replicates (a) is plotted along with the corresponding expression stabilities of six of these genes in three individual biological replicates (b). GeNorm M values of less than 0.5 represent genes with high reference target stability. Please consult Supplementary Table S1 for further details regarding the tested reference genes.

Figure S3 | Heat map of normalized gene expression in Beja 1 root (left) and nodule (right) tissue. TPT values were log_2_ transformed, and subsequent hierarchical clustering of genes was performed employing Euclidean distance calculations. The clusters were consolidated by average linkage. Please consult Supplementary Table S1 for an accordingly sorted list of the genes.

Figure S4 | MapMan-based functional analysis of important regulation pathways (a), primary metabolism (b), and cellular response pathways in relation to biotic and abiotic stress (c). Please consult Figure 4 for further details.

Figure S5 | Alignment of the Mtst1 mRNA sequence from *M. truncatula* and the genomic sequence of the nodule-upregulated monosaccharide transport protein. Calculations were performed with Clone Manager (version 7.11) using the global DNA alignment mode for comparison of two sequences with standard parameter settings.
