# Supplementary material for: Genome-based analysis of the transcriptome from mature chickpea root nodules
Source: Front Plant Sci. 2014 Jul 11;5:325. doi: 10.3389/fpls.2014.00325 (PMC4093793; doi:10.3389/fpls.2014.00325)
Supplement: Supplementary file 1 [file Presentation1.ZIP › Supplementary Figures.docx]

***Supplementary Material***

**Genome-based analysis of the transcriptome from mature chickpea root nodules**

**Fabian Afonso-Grunz^1,2*^, Carlos Molina^2,3^, Klaus Hoffmeier^2^, Lukas Rycak^2^, Himabindu Kudapa^4^, Rajeev Varshney^4^, Jean-Jacques Drevon^5^, Peter Winter^2^, Günter Kahl^1,2^**

^1^ Institute for Molecular BioSciences, Goethe University Frankfurt am Main, Frankfurt am Main, Germany

^2^ GenXPro GmbH, Frankfurt Biotechnology Innovation Center (FIZ), Frankfurt am Main, Germany

^3^ Plant Breeding Institute, Christian-Albrechts-University Kiel, Kiel, Germany

^4^ International Crops Research Institute for the Semi-Arid Tropics (ICRISAT), Hyderabad, Andhra Pradesh, India

^5^ French National Institute for Agricultural Research (INRA), Montpellier-Cedex, France

*** Correspondence:** Fabian Afonso-Grunz, Laboratory of Prof. Dr. Günter Kahl, Institute for Molecular BioSciences, Goethe University Frankfurt am Main, Max-von-Laue-Str. 9, 60438 Frankfurt am Main, Germany.

FGrunz@rz.uni-frankfurt.de

**Supplementary Figures**

Figure S1 Results of the functional classification for the reference mapping file using Mercator.

Figure S2 Reference target stability of selected candidate genes according to geNorm.

Figure S3 Heat map of normalized gene expression in Beja 1 root (left) and nodule (right) tissue.

Figure S4 MapMan-based functional analysis of important regulation pathways, primary

metabolism, and cellular response pathways in relation to biotic and abiotic stress.

Figure S5 Alignment of the Mtst1 mRNA sequence from *M. truncatula* and the genomic sequence of the nodule-upregulated monosaccharide transport protein.


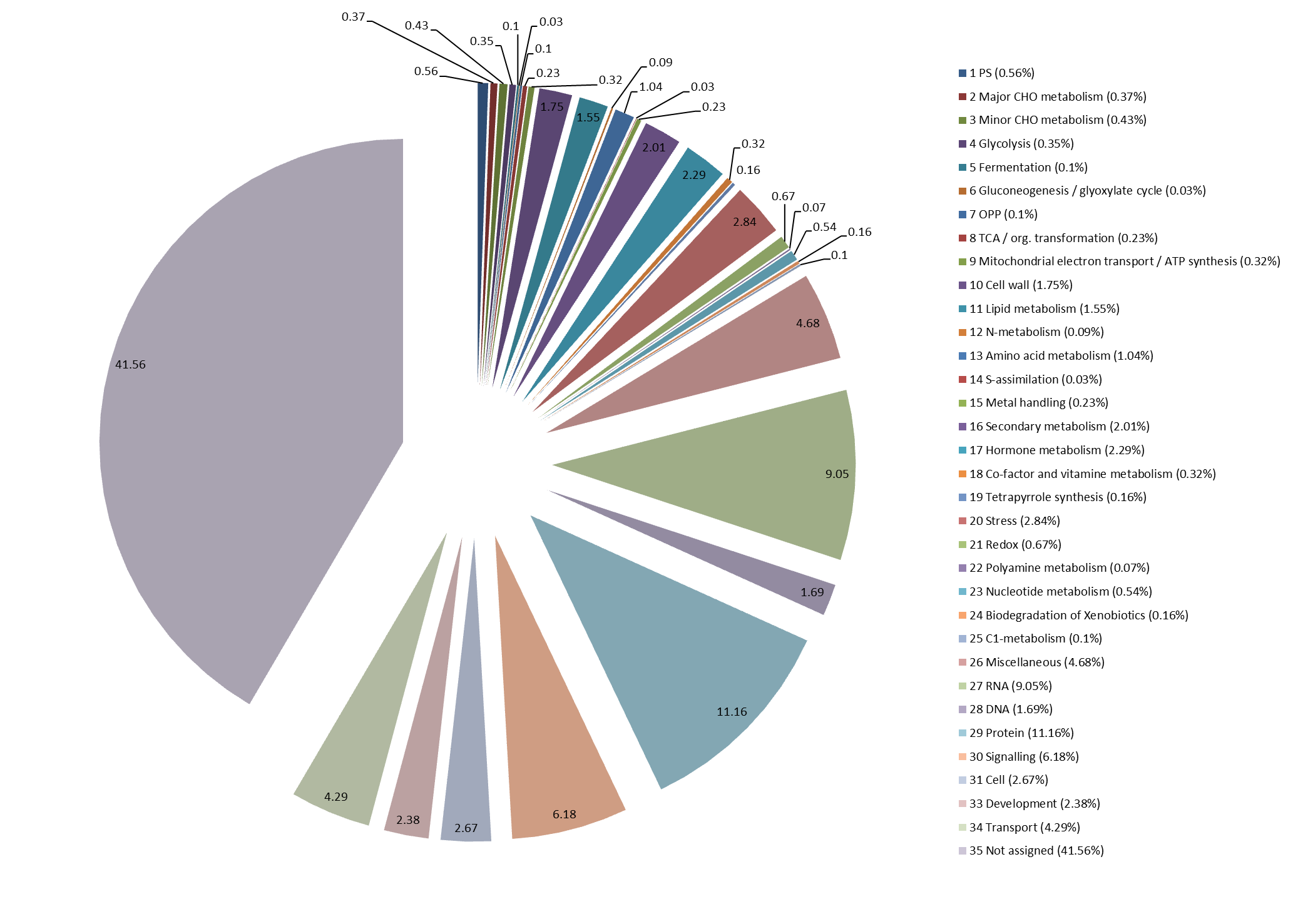


**Figure S1** | **Results of the functional classification for the reference mapping file using Mercator.** The percentage of assigned reads for each of the 34 functional classes is listed and depicted in a circle graph along with the numbers of unassigned reads.


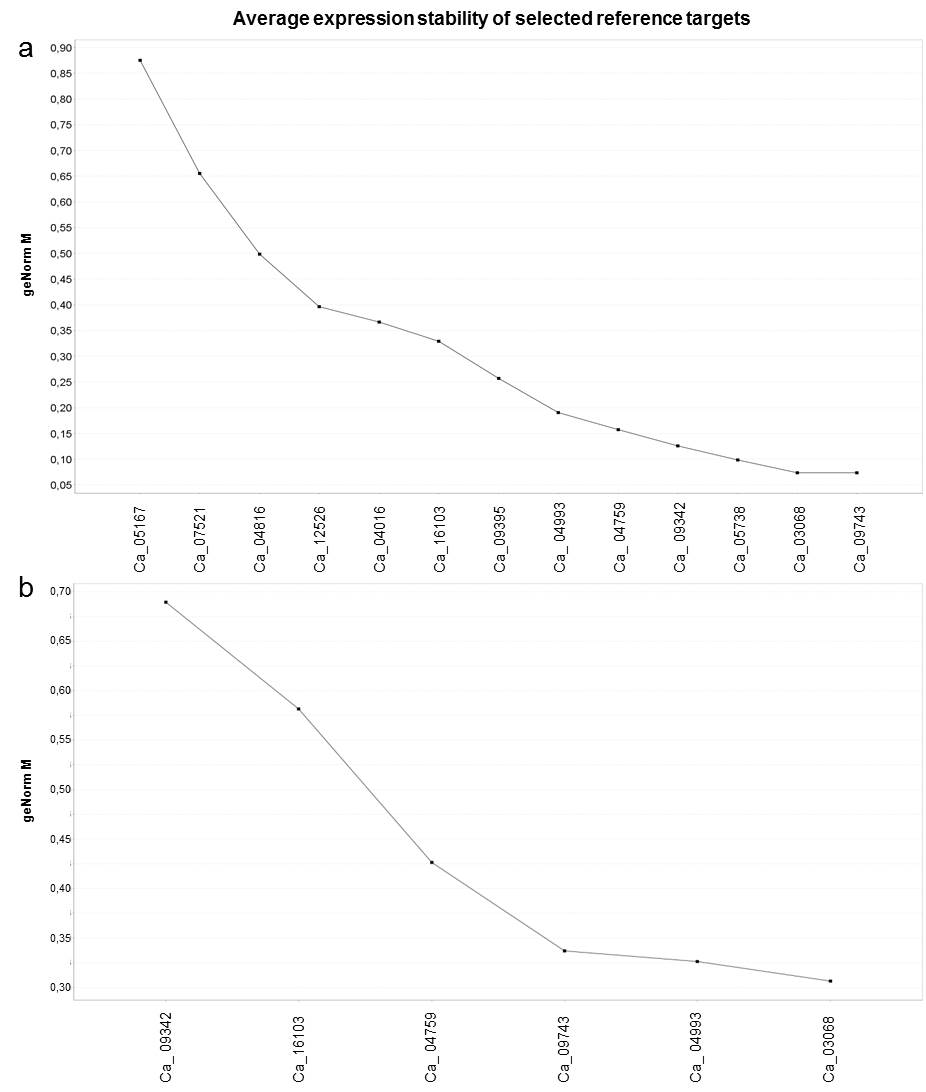


**Figure S2 | Reference target stability of selected candidate genes according to geNorm.** The average expression stability (geNorm M) of 13 potential reference genes in a pool of reverse-transcribed cDNAs from root and nodule tissue of five biological replicates (**a**) is plotted along with the corresponding expression stabilities of six of these genes in three individual biological replicates (**b**). GeNorm M values of less than 0.5 represent genes with high reference target stability. Please consult Supplementary Table S1 for further details regarding the tested reference genes.


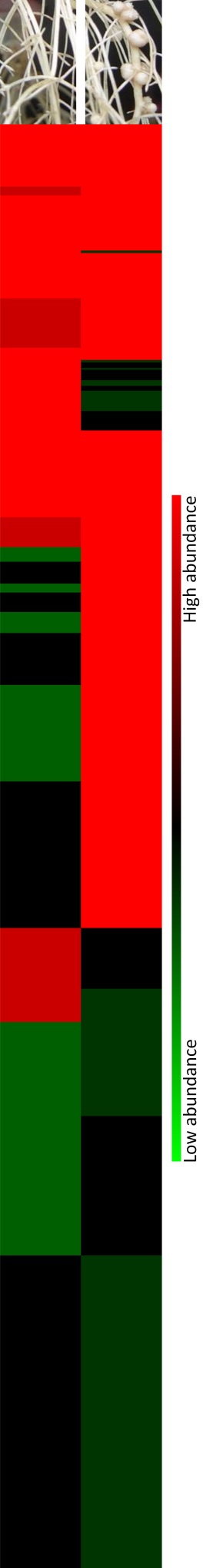


**Figure S3 | Heat map of normalized gene expression in Beja 1 root (left) and nodule (right) tissue.** TPT values were log_2_ transformed, and subsequent hierarchical clustering of genes was performed employing Euclidean distance calculations. The clusters were consolidated by average linkage. Please consult Supplementary Table S1 for an accordingly sorted list of the genes.


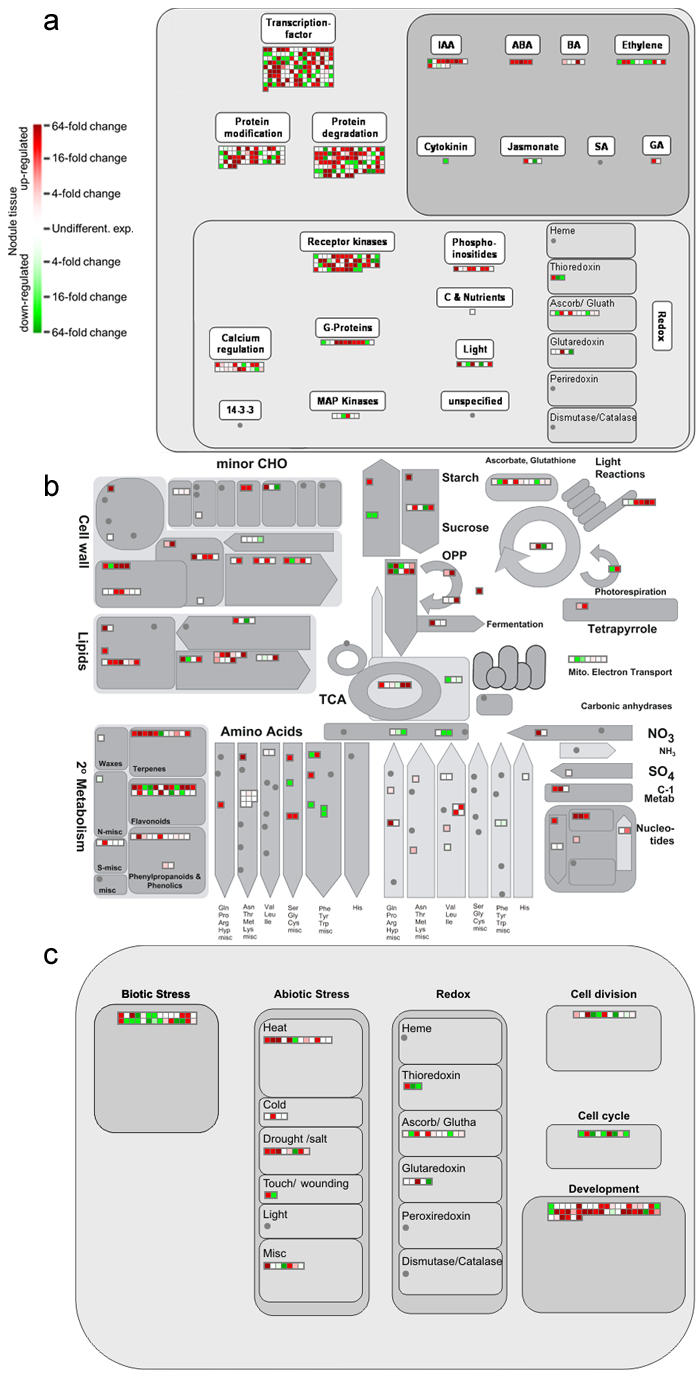


**Figure S4 | MapMan-based functional analysis of important regulation pathways (a), primary metabolism (b), and cellular response pathways in relation to biotic and abiotic stress (c).** Upregulated genes in nodule tissue are shown in red, genes with reduced expression in green, and undifferentially expressed ones in white. Dark gray fields indicate that none of the expressed genes could be assigned to the respective class.

Mtst1 1,753 bp *Medicago truncatula* sugar tranporter mRNA sequence

(GenBank accession: U38651.1)

MTP 2,854 bp genomic sequence of the differentially expressed monosaccharid transport protein on chromosome 6 (Gene ID: Ca_22023)

Mtst1 1 cttcttcttgctactgaggtcagaaaaaatggctggtggtggaattcccattggagggggtaacaaagagtaccccggaa

MTP 1 ----------------------------atgcctgctgtaggaatacccatcggaggtgggaacaaggagtatcccggaa

Mtst1 81 acctcactccttttgtcaccataacatgcatcgttgctgccatgggtggtttgatctttggctacgatattggaatt---

MTP 53 acctcactccttttgtaaccgtaacatgcatcgtggctgccatgggtggtttgatcttcggctacgatattgggatttca

Mtst1 --------------------------------------------------------------------------------

MTP 133 ggtacataaatcatacatactacttggatattgattcttttttgtttgagcgtattataagttttgattttgttgaatgg

Mtst1 158 -------tcaggtggtgtgacgtccatggatccgtttctgaagaaattttttccggcggtgtaccggaaaaagaacaagg

MTP 213 gttttgatcaggtggagtgacgtctatggatccgtttttaaagaagttttttccggcggtatacagaaagaaaaacgatg

Mtst1 231 acaaatcgacaaaccagtactgtcaatatgacagtcaaacattgacgatgtttacatcgtcgttgtatctggctgccctt

MTP 293 acaaaacgacaaaccaatactgtcaatacgacagtcaaacactgacaatgtttacgtcgtcgctttaccttgctgcgtta

Mtst1 311 ttgtcctcgttggtagcttccaccataactcgtaggtttggtcggaaactttccatgcttttcggaggattacttttcct

MTP 373 ctgtcgtcgttggtggcttccaccgttactcgtaagtttggtagaaaactttccatgcttttcggaggtcttcttttcct

Mtst1 391 tgtcggtgctcttattaatggctttgctaatcatgtttggatgttgatcgtgggtcggatcttgctcgggtttggtatcg

MTP 453 tgttggtgctcttattaatggctttgcacaacaagtttggatgttgattgtcggtcggatcttgctcggctttggtatcg

Mtst1 471 ggtttgctaatcagc-----------------------------------------------------------------

MTP 533 gttttgcaaatcaggtactcttttttcttttcctctgttttcttttttatttctttatttttttttttttttgaaaacgg

Mtst1 --------------------------------------------------------------------------------

MTP 613 tttaacacaaaatatgggtgagtctgtatacttccacttttttagcgtacagcaaatttggaatgattcaaattcagact

Mtst1 --------------------------------------------------------------------------------

MTP 693 tgtttgcgtttgattccttcattgaagttttatcctcttttttctttaaataataattaaaaagtaatgaatatgtctgt

Mtst1 --------------------------------------------------------------------------------

MTP 773 tgtcctttaatttgtctgttttgcactattttaattcaccttagttttgatcataaataaaaattacttgaaggaaaaaa

Mtst1 --------------------------------------------------------------------------------

MTP 853 ttttaaaaaattacaagaattaaaaaaattgttagttgtattaattttttgagaataaatattgtttttcatgtttatat

Mtst1 --------------------------------------------------------------------------------

MTP 933 gtgaagagatgattagtgaataatatttttataatatttattgttggttgttgaaatgtaaattagagatatttaagaaa

Mtst1 --------------------------------------------------------------------------------

MTP 1013 aagaaataattaataataaattaataaatgtaaattattgtacaggctttttgaaattttctttcctgcttaaatatggt

Mtst1 --------------------------------------------------------------------------------

MTP 1093 attttttaaaattattgttataatgtatgtcgtaactagttctttgctttttgtgtgtgtttaattgaaagtaaactctt

Mtst1 --------------------------------------------------------------------------------

MTP 1173 tctctataaagtatactgactcatagactcatatagtaaggtgttacaaaaaatttatggcattaattgaattaattaat

Mtst1 --------------------------------------------------------------------------------

MTP 1253 gagtattttatttttttctgaaagtggctgacgtaattaattggtgggtaacgtggattttgattttttccaccttagga

Mtst1 --------------------------------------------------------------------------------

MTP 1333 tttgatttgatctttgcgttctgaaacttttgacttgtttggttgcggtgaaaataaaaggaatcttggccttatttttc

Mtst1 --------------------------------------------------------------------------------

MTP 1413 ttttcgctatcatagacatagtttgagtgtgatcgcttgatttgaacacaatttgatagtttggcactttgctctcacta

Mtst1 --------------------------------------------------------------------------------

MTP 1493 atttaaatattatatagtgccataattattgtgccccgtttttcattccatgtgatgatatgcatcattttccacatcct

Mtst1 486 ----------------------------------------------------------------------------ctgt

MTP 1573 ttgcatttgaaatatttaattaattattcttaattgacttaatttgttaattattaattatttaaaatttattaggctgt

Mtst1 490 gccattgtacctctctgagatggctccttacaagtatagaggagcattgaatattgggtttcaattatcaattacaattg

MTP 1653 gccactgtacctctctgagatggctccatacagatatagaggagctttgaacattggatttcaattatcaattacaattg

Mtst1 570 gtatacttgtggccaatgtgttgaattacttttttgccaaaatcaaaggtggatggggatggagattgagtttaggtggt

MTP 1733 gtatacttgtggccaatgtgttgaactacttttttgccaaaatcaaaggaggttggggatggagattaagtttgggtgga

Mtst1 650 gctatggtcccagcacttataataacaattggatcattagtccttcccgacacccctaactcaatgatcgaacgtggtga

MTP 1813 gctatggttcctgctcttataataactattggatcattagtccttcctgacacccccaattctatgattgaacgtggtga

Mtst1 730 tcgcgatggagctaaagctcaacttaagagaattcgcggcattgaagatgttgatgaagagtttaatgacctcgtagcag

MTP 1893 tcgtgatgctgctaaaattcaacttaagaaaattcgtggtgttgaagatgttgacgaagagtttaatgatcttgttgctg

Mtst1 810 ctagtgaggcctcaatgcaagttgaaaacccttggagaaatttgttgcagaggaaatatagacctcagcttactatggct

MTP 1973 ctagtgaagcttccatgcaagttgaacacccttggaggaacttgttgcaaaggaaatatagacctcaacttactatggct

Mtst1 890 gtattgataccattcttccaacaatttacaggcatcaatgttatcatgttttatgcacctgtgctatttaattccattgg

MTP 2053 ataatgattccctttttccaacaatttactggaattaatgttattatgttttatgcacctgtcctctttaattctattgg

Mtst1 970 gtttaaggacgatgcttcacttatgtcggctgtcatcaccggtgttgttaatgttgttgctacttgtgtctcaatttatg

MTP 2133 atttaaggatgatgcttctcttatgtctgctgtcatcactggtgttgttaatgttgttgctacttgtgtctctatttatg

Mtst1 1050 gagttgataagtggggtaggagagcccttttccttgaaggtggtgctcaaatgctcatatgccagg--------------

MTP 2213 gtgttgacaagtggggtaggagagctctttttcttcaaggtggagctcaaatgatcctatgtcaggtacacttcttttaa

Mtst1 1116 -ttgcagta-----------------------------------------------------------------------

MTP 2293 ttttcattatggttgtaaataatattgtttgcttttatactaattacatcaacttttgttgactcaacgttctcttatat

Mtst1 1124 -----------------gcagctgcaattggggccaaatttggaacaagtggaaaccctggtaatttaccagaatggtat

MTP 2373 gtgtgcaggtcgtagttgcagctgctattggggccaagtttggagttgatggaaatcctggtgatttaccaaagtggtat

Mtst1 1187 gctatagtagttgtgctcttcatttgcatttacgtagcaggatttgcttggtcatggggtcctcttggttggttggttcc

MTP 2453 gctgtagtagttgtgctcttcatttgcatttatgtagcaggatttgcttggtcatggggtcctctaggttggttggtgcc

Mtst1 1267 tagtgagattttcccattggagattcgttctgcagctcaaagtgtaaacgtatctgtgaacatgcttttcaccttcttag

MTP 2533 tagtgagatttttccattggagattcgttccgctgctcaaagtatcaatgtgtcggttaacatgctcttcaccttctttg

Mtst1 1347 ttgcacaagttttcttgataatgctttgtcacatgaagtttggtttgttcctcttctttgccttcttcgttttggtgatg

MTP 2613 ttgcacaaattttcttgacaatgctttgtcacatgaagtttggcttgtttatcttcttcgcggcgtttgttgtggtgatg

Mtst1 1427 tcaatctatgtattcttcttattgcctgaaactaaaggaataccaattgaagagatggacagagtttggaaatcacatcc

MTP 2693 acgatatatatatacttcatgttgcctgagactaagggaataccaattgaagagatgactagggtttggaaatcacatcc

Mtst1 1507 cttctggtctagatttgttgaaca---tggtgatcatggcaatggtgttgagatgggaaagggagctcctaaaaatgtgt

MTP 2773 atattggtctaaatatgtggaacacggtgatgattatggcaatggtgttgagatgggcaagggagctgttaaaaatgtg-

Mtst1 1584 aattattattattagtcttcattttattttattttcattattaattagttttattggtgaaacactaactattggtgtca

MTP --------------------------------------------------------------------------------

Mtst1 1664 acctcaagtatcaaatgtaatgaaattgcacttcaaaattacgggattatttttctcaaaaaaaaaaaaaaaaaaaaaaa

MTP 2852 -----------------taa------------------------------------------------------------

Mtst1 1744 aaaaaaaaaa

MTP ----------

*Total length of aligned sequences with gaps: 3050 bps*

1361 out of 3050 (45%) matching bases

1493 out of 3050 (49%) gaps

196 out of 3050 (6%) mismatches

**Figure S5 | Alignment of the Mtst1 mRNA sequence from *M. truncatula* and the genomic sequence of the nodule-upregulated monosaccharide transport protein.** Calculations were performed with Clone Manager (version 7.11) using the global DNA alignment mode for comparison of two sequences with standard parameter settings.
